# SMOOTHING SPLINE ANOVA MONITORING PHYSICAL ACTIVITY AND FALL RISK IN COMMUNITY-DWELLING OLDER ADULTS

**DOI:** 10.1093/geroni/igad104.2820

**Published:** 2023-12-21

**Authors:** Rui Xie, Lulu Chen, Joon-Hyuk Park, Jeffrey Stout, Ladda Thiamwong

**Affiliations:** University of Central Florida, Orlando, Florida, United States; University of Central Florida, Orlando, Florida, United States; University of Central Florida, Orlando, Florida, United States; University of Central Florida, Orlando, Florida, United States; University of Central Florida, Orlando, Florida, United States

## Abstract

Emerging sensing techniques, which are easily transportable, user-friendly, and cost-effective, may reveal new possibilities in monitoring daily objectively measured physical activity (PA) and fall risk appraisal (FRA) identification. We used a Smoothing Spline Analysis of Variance (SS-ANOVA) to profile the daily PA using accelerometer-based device across FRA groups. Using the BTrackS system (static balance assessment) and a short Fall-Efficacy Scale-International, 124 participants (mean age 75, range 60-96, 77% female, 72% no history of falls) were categorized into four different FRA groups: rational (46.8%, low fear of falling (FOF) and normal balance), irrational (17.7%, high FOF and normal balance), incongruent (19.4%, low FOF and poor balance), and congruent (16.1%, high FOF and poor balance). The vector magnitude (VM) and the number of steps were extracted per second from an average 10 (SD=4.89) consecutive days of PA recording. Daily (0-24h) temporal trend was analyzed from 1.7 million records. Groupwise, PA of the congruent group was significantly lower than other three groups, while the rational group had the highest PA based on 95% Bayesian C.I. It is evident that there is a periodic trend in PA; in particular, inactive during night (12-5am), increasing trend in the morning (5am-noon) and peak at noon, moderately decrease in the afternoon (noon-6pm), and significantly decline in the evening. No significant gender differences in PA were found. These results indicate that FOF associated with poor balance contributes to low PA, highlighting the necessity of balance enhancing intervention and cognitive reframing to promote PA in older populations.

